# Neural Network-Based Multi-Objective Optimization of Adjustable Drawbead Movement for Deep Drawing of Tailor-Welded Blanks

**DOI:** 10.3390/ma15041430

**Published:** 2022-02-15

**Authors:** Parviz Kahhal, Jaebong Jung, Yong Chan Hur, Young Hoon Moon, Ji Hoon Kim

**Affiliations:** 1 School of Mechanical Engineering, Pusan National University, Busan 46241, Korea; parvizkahhal@abru.ac.ir (P.K.); sylar999@pusan.ac.kr (J.J.); kainpower@pusan.ac.kr (Y.C.H.); yhmoon@pusan.ac.kr (Y.H.M.); 2 Department of Mechanical Engineering, Ayatollah Boroujerdi University, Boroujerd 6919969737, Iran

**Keywords:** multi-objective optimization, tailor-welded blank sheets, artificial neural networks, genetic algorithm, forming limit diagram

## Abstract

To improve the formability in the deep drawing of tailor-welded blanks, an adjustable drawbead was introduced. Drawbead movement was obtained using the multi-objective optimization of the conflicting objective functions of the fracture and centerline deviation simultaneously. Finite element simulations of the deep drawing processes were conducted to generate observations for optimization. The response surface method and artificial neural network were used to determine the relationship between variables and objective functions; the procedure was applied to a circular cup drawing of the tailor-welded dual-phase steel blank. The results showed that the artificial neural network had better prediction capability and accuracy than the response surface method. Additionally, the non-dominated sorting-based genetic algorithm (NSGA-II) could effectively determine the optima. The adjustable drawbead with the optimized movement was confirmed as an efficient and effective solution for improving the formability of the deep drawing of tailor-welded blanks.

## 1. Introduction

Sheet metal forming is an important production method in various industries. In this process, a sheet blank is transformed into a product with a complex shape and desirable engineering properties using tools. Tailor-welded blanks (TWBs) consist of two or more sheets whose material, thickness, and surface coating can be similar or different and are joined before the forming process. TWBs can reduce product weight, material consumption, and costs while improving strength [1]. With TWB sheets, understanding their formability has become essential for producing high-quality products in forming processes.

Many studies have been conducted on TWB forming. TWBs were first used to overcome design challenges with existing materials, such as the Audi 100 floor panel [2,3]. Chatterjee et al. [4] evaluated the formability and mechanical behavior of dual-phase TWBs; they discussed the effect of weld orientation on the formability of TWBs. Xia et al. [5] investigated the formability of high-strength low-alloy (HSLA) steel TWBs welded using diode lasers.

If sheets with dissimilar flow properties are used in TWBs, the deformation of TWBs during the drawing process can be more complex than that of homogeneous sheets; in particular, the weld line typically moves toward a harder material. Some methodologies have been proposed to control the movement of the weld line, such as controlling the blank holder forces (BHFs), introducing drawbeads, and constraining the weld line. Kinsey et al. [6] proposed a clamping method to reduce the maximum strain along the weld. Heo et al. [7] studied the effect of drawbead movement of the weld line and thickness strain distribution on square TWBs. He et al. [8] used a BHF control strategy to improve the forming capability of TWBs. Padmanabhan et al. [9] investigated the effect of a non-uniform BHF on the punch force, thickness variation, and movement of the weld line in the drawing of TWBs made of dissimilar materials. Korouyeha et al. [10] investigated the forming limit diagram and forming capability of TWBs based on thickness ratio variation. Hu et al. [11] analyzed the movement of the weld line, wrinkling, and thinning ratio to gauge the formability of TWBs. Suresh et al. [12] reduced the weld line movement by using a special punch that included two parts separated by an isolating material; one part was used for heating the thicker (or stronger) sheet, and the other was used for cooling the thinner (or weaker) one. They investigated the thickness ratios’ influence on the weld line’s movement at different temperatures. Gautam and Kumar [13] studied the formability of TWBs with high-strength steel JSC590RN parent sheets.

The finite element method (FEM) is successfully applied in numerous manufacturing fields as a powerful tool to reduce experimental time and costs [14,15]. About TWBs, Wu et al. [16] proposed a finite element model to investigate the effect of thickness on the limiting dome height (LDH) testing of aluminum TWBs. It was found that increasing thickness would decrease the normalized equivalent plastic strain and normalized LDH values. Heo et al. [17] investigated how the drawbead dimensions affect the movements of the weld line during the forming of TWBs. Kumar and Digavalli [18] numerically evaluated the formability of low-carbon steel TWBs with two different thicknesses in hydraulic bulging. It was found that with an increase in the thickness ratio, the peak pressure increases, and simulation results will be closer to experimental ones. Padmanabhan et al. [19] studied springback in forming TWBs under various stress states. Aminzadeh et al. [20] developed an approach to predict residual stresses in TWBs using FEM followed by experimental validation. Kridli et al. [21] investigated the formability of aluminum TWBs using a limiting dome height test. Asadian-Ardakani et al. [22] investigated the deep drawing process of interstitial-free steel TWBs with non-uniform blank holder forces, both theoretically and experimentally. Panda et al. [23] investigated the tensile properties of steel TWBs and their formability in stretch forming.

Adjustable drawbeads have been introduced to control the formability of sheet metal blanks and TWBs [24]. Li and Weinmann [25] used selected drawbead trajectories to investigate formability in non-symmetric aluminum panel drawings. Li et al. [26] later used a set of drawbead trajectories to optimize the maximum attainable draw depth at the fracture. Wang et al. [27] used variable BHF to improve the thickness distribution and optimize the forming capability of aluminum alloy sheet panels on automobiles.

In recent years, research has been conducted on optimizing the process parameters for forming TWBs. Dhumal et al. [28] investigated the forming behavior of TWBs using an artificial neural network (ANN) to predict the maximum draw depth and weld line profile. Hariharan et al. [29] optimized the drawbead geometry to achieve minimum movement of the weld line and maximum dome height.

When the actual relationship between the input and output is unknown or computationally expensive to evaluate, surrogate models are used to map input data to output data. Famous surrogate modeling methods are the response surface model (RSM), the Bayesian approaches, the radial basis function (RBF), the support vector machines (SVM), and the artificial neural networks (ANN) [30]. The RSM is one of the most popular techniques used for regression processes. The ANNs are powerful and flexible tools well suited to modeling complex and non-linear processes.

Various optimization algorithms are used such as the strength Pareto evolutionary algorithm (SPEA), the multi-objective particle swarm optimization (MOPSO), the multi-objective ant colony optimization (MOACO), and the fast non-dominated sorting genetic algorithm (NSGA-II). Among them, the advantages of NSGA-II are as follows:

1. It uses non-dominated sorting techniques to provide as close to the Pareto-optimal solution as possible.
2. It searches for all non-dominated solutions (F1) considering the crowding distance, which helps obtain diverse results.
3. It also uses elitist techniques to preserve the best solution for the current population in the next generation [31].

For these advantages, NSGA-II has been regarded as one of the most studied algorithms for multi-objective optimization [32].

In this study, an adjustable drawbead was used to improve the formability of TWBs. The trajectories of the adjustable drawbead were considered as the optimization variables. The fracture and weld line movements were optimized simultaneously as formability objectives using a non-dominated sorting-based genetic algorithm (NSGA-II). Designing of experiments was conducted using two methods: response surface model (RSM) and ANN; the accuracy of the two designs was also discussed.

## 2. Deep Drawing Process of Tailor-Welded Blanks

**Figure 1:**
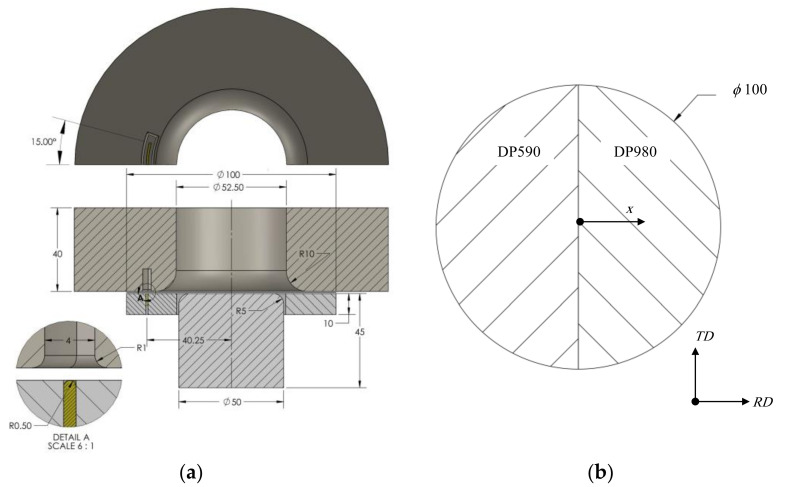
(**a**) Section view of cylindrical cup drawing tools, and (**b**) tailor-welded blank sheet (unit: mm).

A cylindrical cup drawing process was used to investigate the effect of the process parameters on the formability of the TWBs. For the TWB, laser-welded dual-phase steels with tensile strengths of 590 MPa and 980 Mpa were used, which were denoted as DP590 and DP980, respectively. The base sheets were supplied by POSCO (Pohang, South Korea), and the chemical compositions are listed in Table 1 [33]. Figure 1 shows the dimensions of the drawing process and the blank shape. A punch speed of 25 mm/s and a blank holder force of 10 kN was used, and the punch stroke was 25 mm.

**Figure 2:**
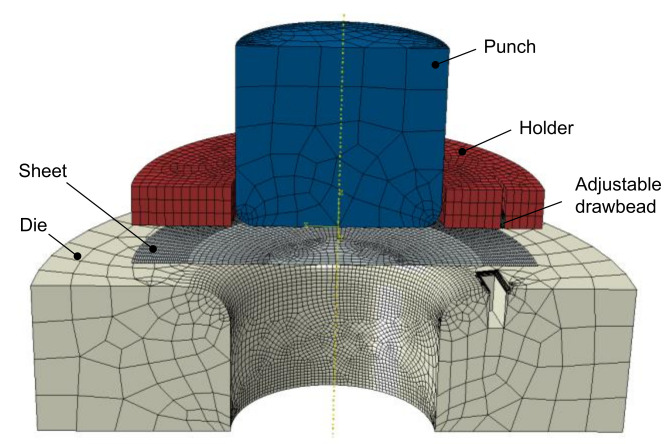
Finite element model of the cup drawing process.

Figure 2 shows the finite element model of the cup drawing process. The die, punch, holder, and bead were modeled using rigid quadrilateral elements (R3D4). In addition, the blank was modeled using 3001 four-node shell elements (S4R) with five integration points through the thickness. Since the hardness of the heat-affected zone was not degraded [34], the fracture is not expected to occur in the weld zone. Therefore, the weld zone was not considered in the finite element model. Surface-to-surface interaction has been defined for die parts—sheet blanks contact with friction coefficient equal to 0.1. Finite element analysis was performed using the ABAQUS/Explicit 6.12 software.

**Figure 3:**
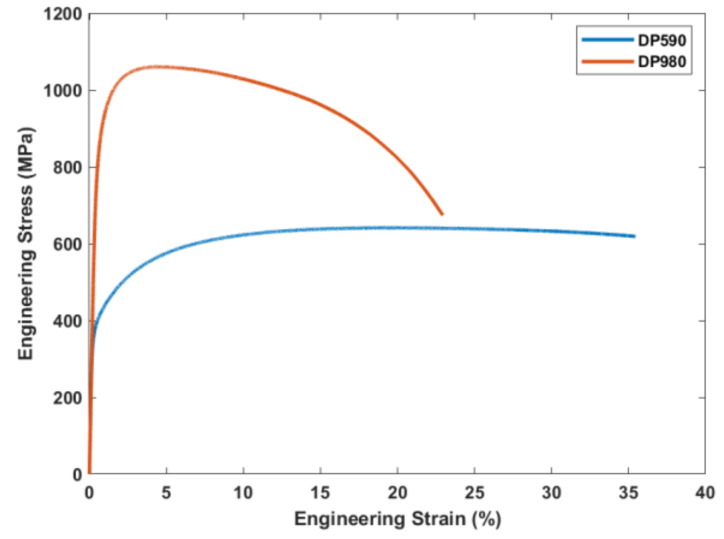
Engineering stress–strain curve for dual-phase steels DP590 and DP980.

The mechanical properties of the base materials of the TWB were determined by tensile tests performed using a universal testing machine [34]. The engineering stress–strain curves of both materials are shown in Figure 3. The mechanical properties of the sheet blank are listed in Table 2.

**Figure 4:**
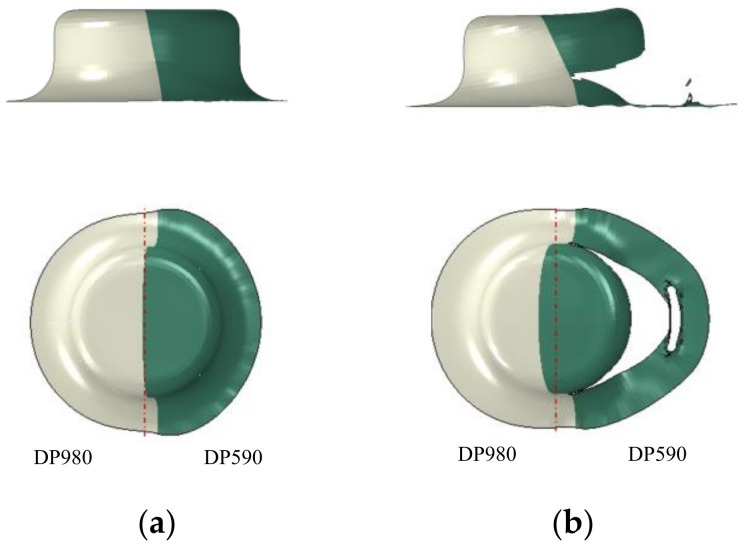
The final shape of drawn blank (**a**) without a bead, (**b**) with fixed bead.

As the two sheets have different mechanical properties, the material on the softer side is drawn in more, and the weld line is pulled away from the center. Without a drawbead, the blank with a lower tensile strength (DP590) flow in more than the blank with a higher tensile strength (DP980), as shown in Figure 4a. A fixed drawbead can control this excessive flow of material, but necking failure may occur on the softer side, as shown in Figure 4b. An adjustable drawbead whose trajectory can be controlled over the punch stroke is introduced to achieve the desired material flow without fracture. By changing the height of the adjustable drawbead as a function of punch displacement, the desired TWB material flow can be achieved without fracture in the deep-drawn cup. A multi-objective optimization procedure can optimize the adjustable drawbead trajectory for achieving these purposes.

## 3. Multi-Objective Optimization

The finite element method (FEM) is a useful tool for reducing experimental costs by simulating metal-forming processes and optimizing process parameters. However, it may be repeated manually with various combinations of process parameters to attain a desirable process. Therefore, a robust and straightforward optimization method is required to enhance the existing design conditions.

**Figure 5:**
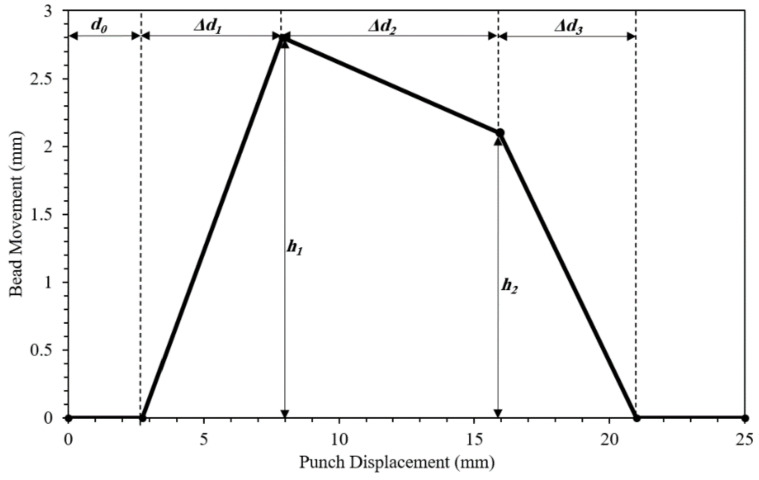
Adjustable drawbead movement as a piecewise linear function of punch displacement.

The purpose of this research is to find the best arrangement of process variables that will result in a tailor-welded part without fracture and excessive flow of materials. The drawbead movement parameters were chosen as the design variables, as shown in Figure 5. The adjustable drawbead movement is defined as a piecewise linear function of the punch displacement. The design variables are $d_{0}$, $\Delta d_{1}$, $\Delta d_{2}$, $\Delta d_{3}$, corresponding to the displacement to the initiation of the bead, displacement to the first peak, displacement to the second peak, and displacement to the termination of the bead; $h_{1}$ and $h_{2}$ are the height of the first and second peaks. All units are in mm.

The non-linear optimization problem can be represented in the following form [35]:

(1) $$\mathrm{Minimize}\;F\left(X\right)=\left(Obj_{f},Obj_{c}\right)\;$$

Subject to:

$$0\leq d_{0}\leq 25$$

$$0\leq \Delta d_{1}\leq 25$$

$$0\leq \Delta d_{2}\leq 25$$

$$0\leq \Delta d_{3}\leq 25$$

$$0\leq h_{1}\leq 3$$

$$0\leq h_{2}\leq 3$$

Constraint:

$$d_{0}+\Delta d_{1}+\Delta d_{2}+\Delta d_{3}\leq 25.$$

### 3.1. Objective Functions

To prevent these defects, measures for defining defects in TWB forming should be defined. The fracture and centerline deviation functions were defined in this study, as explained below.

#### 3.1.1. Fracture

**Figure 6:**
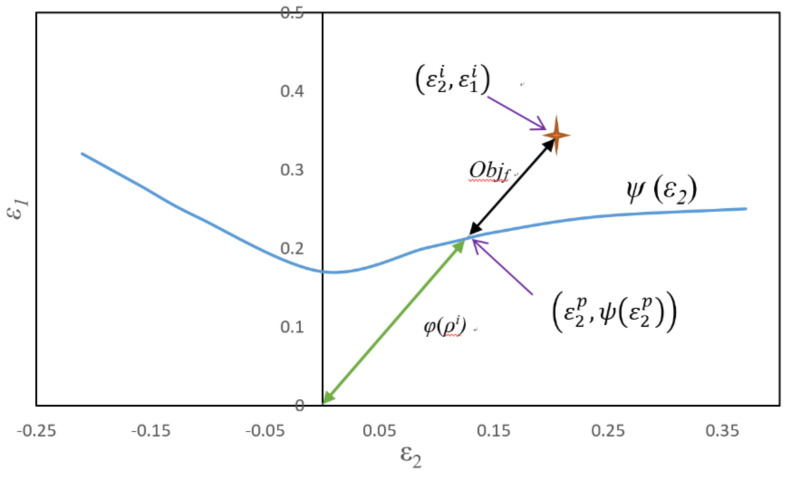
Definition of the fracture objective function based on the forming limit diagram.

When the strains of the elements are above the forming limit curve, fracture occurs, as shown in Figure 6. The objective function of the fracture can be defined as:

(2) $$Obj_{f}=\frac{1}{n}\overset{n}{\underset{i=1}{\sum }}H\left(\sqrt{{\left(\varepsilon_{1}^{i}\right)}^{2}+{\left(\varepsilon_{2}^{i}\right)}^{2}}-\varphi \left(\rho^{i}\right)\right)$$

where *n* is the number of elements, $\varepsilon_{1}^{i}$ and $\varepsilon_{2}^{i}$ are the major and minor strains of element *i*, *H* is the Heaviside function, $\varphi \left(\rho^{i}\right)$ is the forming limit function, and $\rho^{i}$ is the strain ratio (=$\varepsilon_{1}^{i}/\varepsilon_{2}^{i}$) of the *i*^th^ element:

(3) $$\varphi \left(\rho^{i}\right)=\sqrt{{\left(\varepsilon_{2}^{p}\right)}^{2}+{\left(\psi \left(\varepsilon_{2}^{p}\right)\right)}^{2}}.$$

The Marciniak–Kuczynski (MK) method was used to calculate the forming limit curves of the two base materials.

#### 3.1.2. Centerline Deviation

To minimize the centerline deviation, the following objective function is defined as:

(4) $$Obj_{c}=\Delta x,$$

where Δx is the deviation of the center point of the sheet, as shown in Figure 1.

### 3.2. Multi-Objective Optimization Algorithm

Multi-objective optimization involves the simultaneous optimization of several objectives. In the conventional genetic algorithm (GA) [36], an individual population randomly distributed over the design space is evaluated based on fitness. The algorithm maintains the best fit, and a new population using GA operators (mutation and crossover) will be evaluated for their fitness. By repeating the generations, the algorithm searches the design space and finally converges to the global optimum. In the multi-objective genetic algorithm (MOGA), a similar approach is considered, except in this case, the intention is to optimize for a collection rather than a fitness parameter [37]; a Pareto front solution was used to achieve this process. For a minimization problem, point $Obj^{1}\left(x\right)$ dominates point $Obj^{2}\left(x\right)$ if and only if

(5) $$Obj_{i}^{1}\left(x\right)\leq Obj_{i}^{2}\left(x\right)\;i=1,\;2.$$

Further, for at least one *j*, 1 ≤ *j* ≤ 2, satisfying

(6) $$Obj_{j}^{1}\left(x\right)<Obj_{j}^{2}\left(x\right).$$

In other words, $Obj^{1}\left(x\right)$ is a Pareto solution if it is not worse in each of the objectives and better in at least one objective than the $Obj^{2}\left(x\right)$.

**Figure 7:**
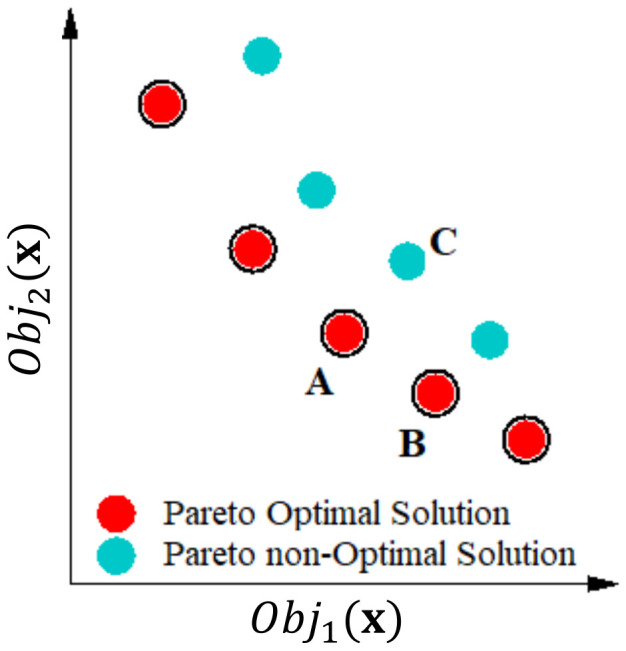
Pareto optimum solutions.

For example, in Figure 7, the red points are the possible choices when considering a minimization problem. At point A, both objectives have smaller values than those of point C. As a result, point A dominates point C. Points A and B cannot dominate each other and therefore are Pareto optimal solutions, and point C is a Pareto non-optimal solution [38].

#### Fast Non-Dominated Sorting Genetic Algorithm (NSGA-II) for Multi-Objective Problems

The fast non-dominated sorting genetic algorithm (NSGA-II) is widely used in multi-objective optimization problems [39]. NSGA-II searches for all non-dominated solutions (F_1_) considering the crowding distance, which helps obtain diverse results. The procedure of NSGA-II is briefly described as follows [40,41]:

Step 1: Population initialization ($P_{t}$).
Step 2: Generate a new population (offspring $Q_{t}$) by applying crossover and mutation to the current population.
Step 3: Combine two populations (individual and offspring) $R_{t}=P_{t}\cup Q_{t}$.
Step 4: Rank the fitness of the new population by employing the non-dominated sorting algorithm and then ordering them as non-dominated fronts $F_{1}$, $F_{2}$, …, $F_{k}$ in $R_{t}$ [42]_._
Step 5: Calculate the crowding distance.
Step 6: Create a new population ($P_{t+1}$) considering rank and crowding distance. When two solutions are in the equivalent rank, the solution with a greater crowding distance is selected.
Step 7: Create offspring population $Q_{t+1}$ by applying crossover and mutation to $P_{t+1}$.
Step 8: Set t=t+1 and go to Step 2.

**Figure 8:**
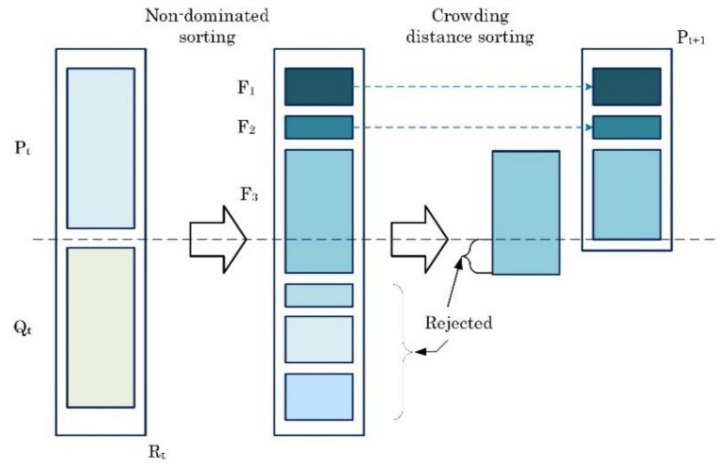
Schematic of NSGA-II procedure.

Figure 8 shows the schematic of the NSGA-II procedure [43].

### 3.3. Design of Experiments

Design of experiments (DOE) is a useful surrogate method to reduce analysis and experimental costs in complex problems; it helps realize the effects of inputs on responses based on statistical calculations. This research used two methodologies to determine the relationship between the design variables and objective function: RSM and ANN-based machine learning. Both methodologies were applied to the model, and the results were compared and discussed.

#### 3.3.1. RSM

RSM is an approach for building approximations of objectives based on observations in the design space. The intensity of this approach is that gradient-based methods fail, i.e., when designing is challenging or impossible to evaluate [44,45]. The choice of surrogate functions to estimate the real performance is crucial. These functions can be defined as polynomials or sums of various basis functions (e.g., sine and cosine).

In this study, a second-order polynomial is used to construct the RSM. If $n_{s}$ analyses are conducted and p = 1; 2; . . .; $n_{s}$, then a second-order polynomial model has the form

(7) $$y^{\left(p\right)}=c_{o}+\underset{1\leq j\leq n_{v}}{\sum }c_{j}x_{j}{}^{\left(p\right)}+\underset{1\leq j\leq k\leq n_{v}}{\sum }c_{\left(n_{v}-1+j+k\right)}x_{j}^{\left(p\right)}X_{k}^{\left(p\right)}$$

where $y^{\left(p\right)}$ is the response, $x_{j}^{\left(p\right)}$ and $X_{k}^{\left(p\right)}$ are the $n_{y}$ design parameters, and $c_{0}$, $c_{j}$, and $c_{\left(n_{v}-1+j+k\right)\;}$ are the unknown coefficients [37].

#### 3.3.2. ANN

**Figure 9:**
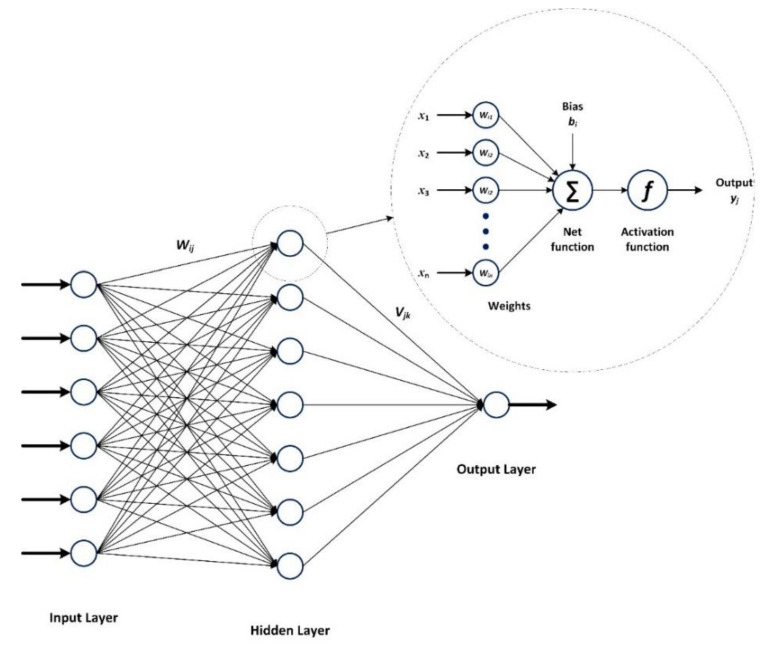
Schematic of MLP and neurons.

An ANN simulates the structure of human synaptic links to represent brain performance. A multilayer perceptron (MLP) is a feed-forward ANN. A typical MLP includes several layers, each containing a group of computational neural cells; each neuron in a layer is connected to other neurons only in neighboring layers. The first layer only contains the values of the input variables, and the last layer calculates the outputs, among which are the hidden layers [46]; each connection line has a weight between the neurons. A multilayer perceptron is trained by modifying these weights (connection weights), with the aim of the computed outputs attaining the desired prediction (Figure 9).

The output of the *j*^th^ neuron in the hidden layer, $y_{j}$, is given by:

(8) $$y_{j}=f_{1}\left(\sum_{i=1}^{n_{m}}\left(W_{ij}x_{i}\right)+b_{j}\right)\;,\;$$

where $f_{1}$ is the activation function in the hidden layer, $n_{m}$ is the number of inputs, $W_{ij}$ is the connection weight between the *i*^th^ neuron in the input layer and the *j*^th^ neuron in the hidden layer, $x_{i}$ is the *i*^th^ input, and $b_{j}$ is the bias assigned to the *j*^th^ neuron in the hidden layer.

The output of an MLP, including one hidden layer, z, can be calculated as follows:

(9) $$z=f_{2}\left(\left(\sum_{j=1}^{n_{n}}V_{j}y_{j}\right)+a\right),\;$$

where $f_{2}$ is the activation function of the hidden layer, *n_n_* is the number of neurons in the hidden layer, $V_{j}$ is the connection weight between the *j*^th^ neuron in the hidden layer and the neuron of the output layer, and *a* is the bias assigned to the output layer.

#### 3.3.3. Sobol Sequence Design

**Figure 10:**
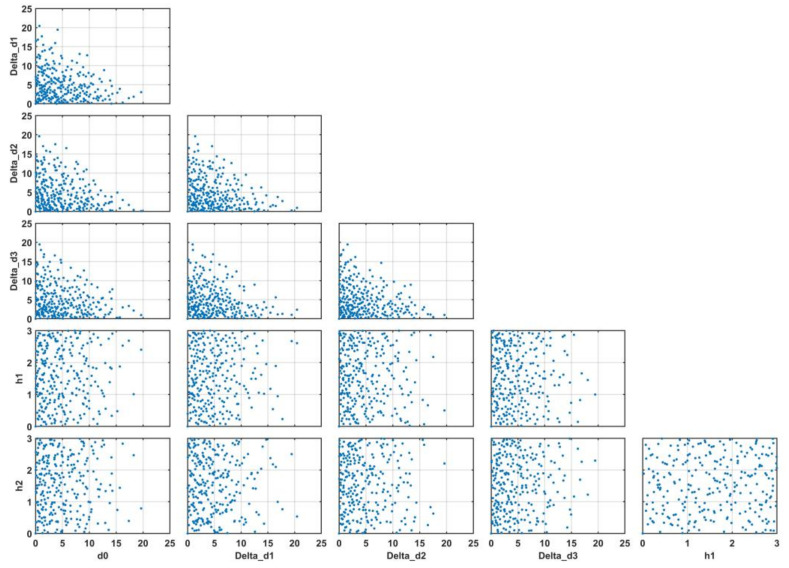
Pairwise projection of variables (Sobol sequence design).

Observations are required to build the RSM and ANN models. These observations (combination of process variables) can be generated by the DOE methods, such as the classical design (e.g., Box-Behnken, full factorial), space filling (e.g., Latin hypercube design, Sobol sequence, Taguchi), and optimal design (e.g., A-optimal, and D-optimal). This study used the space-filling Sobol sequence sampling method [47] to generate 256 sampling points for the DOE. The pairwise projections of the variables are shown in Figure 10.

### 3.4. Optimization Model

**Figure 11:**
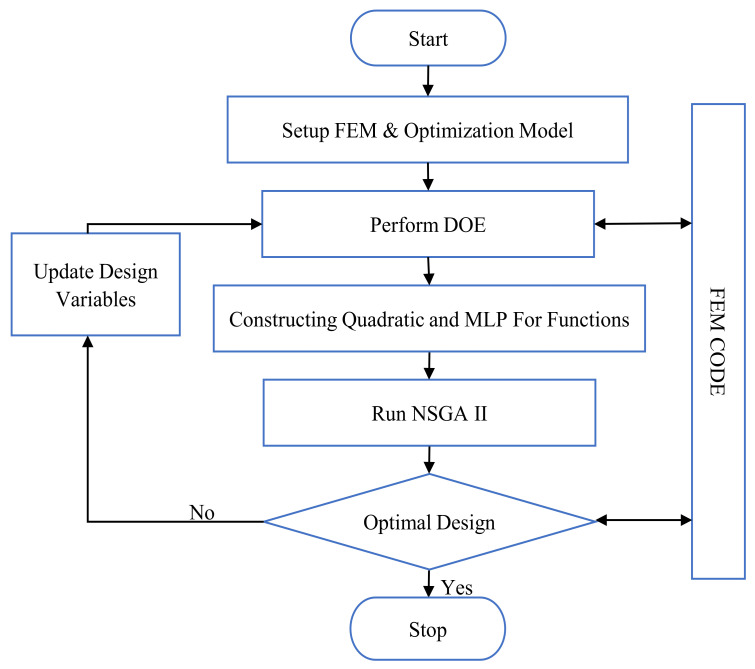
Optimization process diagram.

Figure 11 shows the optimization process diagram, which includes five steps:

Step 1: The finite element and optimization models were initialized, and subsequent parameters were assigned. (1) Population size P = 250, (2) Reproduction: crossover fraction F_c_ = 0.8, mutation fraction F_m_ = 0.1, (3) Stopping criteria: termination generation T = 1000 or stall generations = 200 or function tolerances = 0.00001.
Step 2: To build the RSM and MLP, the objective functions are calculated for each DOE Sobol sequence design observation.
Step 3: According to Equations (7) and (8), the RSM and MLP networks can be created based on the DOE.
Step 4: The NSGA-II algorithm is used to determine the Pareto front. The optimization algorithm uses surrogate models for evaluating the objective function value instead of long-time FEA computation.
Step 5: If the termination criterion is reached, the algorithm is stopped. Otherwise, the process returns to step 3.

## 4. Results

FEA simulations were carried out for all the Sobol sequence design matrix, and objectives were obtained.

### 4.1. RSM and ANN

For RSM, objective functions were obtained as a quadratic polynomial.

(10) $$\begin{array}{l} Obj{\;}_{f}=-7.0855\times 10^{-5}+8.3995\times 10^{-5}d_{0}+2.3874\times 10^{-5}\Delta d_{1}-2.48\times 10^{-5}\Delta d_{2}-\\ 3.4702\times 10^{-5}\Delta d_{3}-0.00014516h_{1}-0.00017348h_{2}-1.3424\times 10^{-6}d_{0}^{2}-7.2703\times 10^{-6}d_{0}\Delta d_{1}\;-\\ 5.4894\times 10^{-6}d_{0}\Delta d_{2}+1.4219\times 10^{-6}d_{0}\Delta d_{3}-3.8064\times 10^{-6}d_{0}h_{1}-1.3781\times 10^{-5}d_{0}h_{2}-\\ 1.5955\times 10^{-6}\Delta d_{1}{}^{2}\;-1.7786\times 10^{-6}\Delta d_{1}\Delta d_{2}+4.8495\times 10^{-7}\Delta d_{1}\Delta d_{3}+4.164\times 10^{-5}\Delta d_{1}h_{1}+\\ 1.5092\times 10^{-6}\Delta d_{1}h_{2}+2.3519\times 10^{-7}\Delta d_{2}{}^{2}+9.3702\times 10^{-7}\Delta d_{2}\Delta d_{3}+2.9055\times 10^{-5}\Delta d_{2}h_{1}+\\ 4.9061\times 10^{-5}\Delta d_{2}h_{2}+2.0589\times 10^{-6}\Delta d_{3}{}^{2}+4.5942\times 10^{-8}\Delta d_{3}h_{1}+5.0094\times 10^{-6}\Delta d_{3}h_{2}-\\ 5.7961\times 10^{-5}h_{1}{}^{2}+8.9244\times 10^{-5}h_{1}h_{2}+6.1988e\times 10^{-5}h_{2}{\;}^{2} \end{array}$$

(11) $$\begin{array}{l} Obj{\;}_{c}=1.2063+0.079381d_{0}-0.0059661\Delta d_{1}-0.055087\Delta d_{2}-0.015468\Delta d_{3}-0.50206h_{1}-\\ 0.33678h_{2}-0.0048769d_{0}^{2}+0.00034024d_{0}\Delta d_{1}+0.0030268d_{0}\Delta d_{2}+0.0013313d_{0}\Delta d_{3}+\\ 0.025424d_{0}h_{1}+0.016668d_{0}h_{2}+0.0010876\Delta d_{1}{}^{2}+0.0036992\Delta d_{1}\Delta d_{2}+0.0013547\Delta d_{1}\Delta d_{3}\;-\\ 0.011031\Delta d_{1}h_{1}+0.013001\Delta d_{1}h_{2}+0.0014909\Delta d_{2}{}^{2}+0.00058581\Delta d_{2}\Delta d_{3}-0.006405\Delta d_{2}h_{1}+\\ 0.0053715\Delta d_{2}h_{2}-0.00014949\Delta d_{3}{}^{2}-0.00092006\Delta d_{3}h_{1}+0.0022475\Delta d_{3}h_{2}+0.063539h_{1}{}^{2}+\\ 0.041187h_{1}h_{2}+0.010835h_{2}{}^{2} \end{array}$$

For ANN, an ANN was trained for each objective function using the observations. The best numbers of neurons and layers were obtained based on grid search. Prescribed combinations of parameters were tested, and the best combination was found. The criteria for the best combination were the minimum standard deviation of the residuals (RMSE) for training (70%), testing (15%), and validation (15%) data. Table 3 lists the parameters of the trained ANN. The number of neurons and layers were chosen for the best predictive capability without underfitting and overfitting.

**Figure 12:**
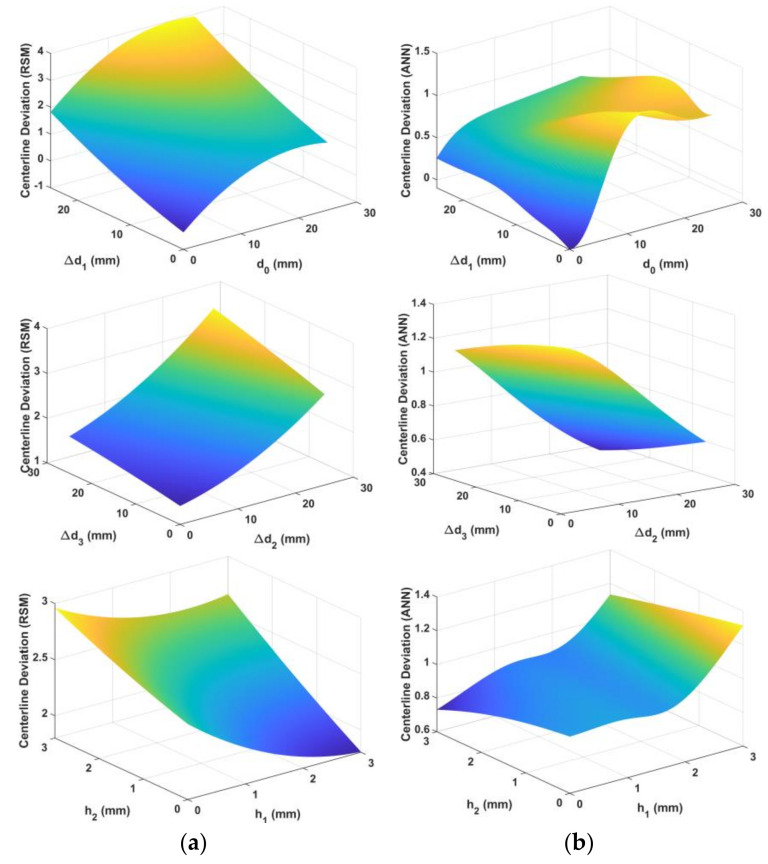
Predicted surface for centerline deviation objectives based on design variables: (**a**) quadratic response surface, (**b**) artificial neural network.

**Figure 13:**
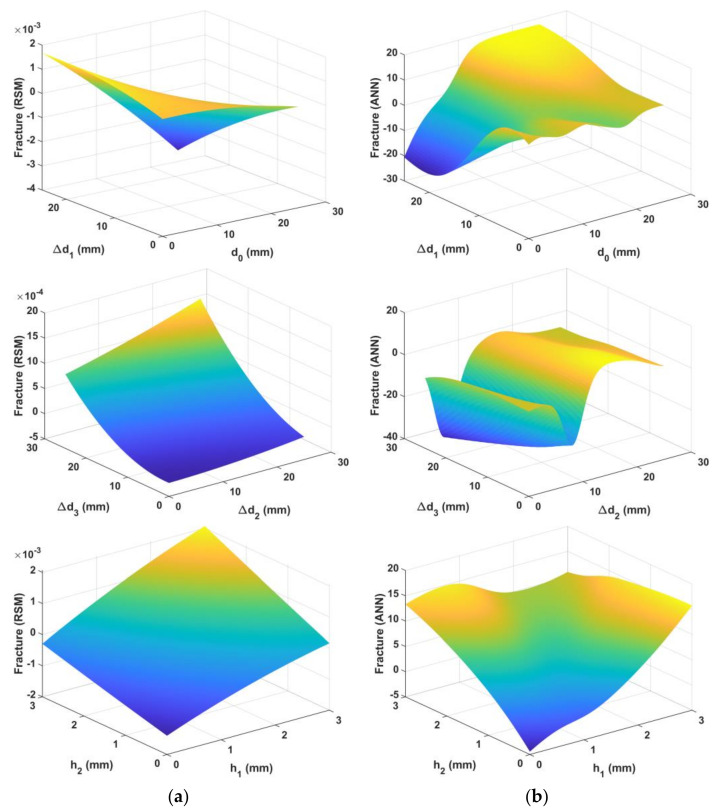
Predicted surface for fracture objectives based on design variables: (**a**) quadratic response surface, (**b**) artificial neural network.

Figure 12 and Figure 13 show the predicted surfaces of the centerline deviation and fracture objectives based on the design variables, respectively.

**Figure 14:**
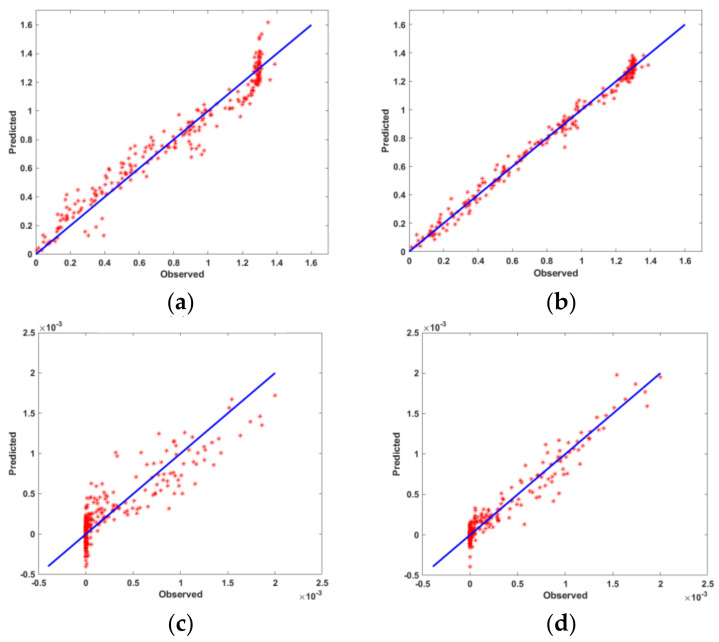
Predicted vs. observed diagram for centerline deviation: (**a**) RSM, (**b**) ANN, and fracture: (**c**) RSM, (**d**) ANN.

**Figure 15:**
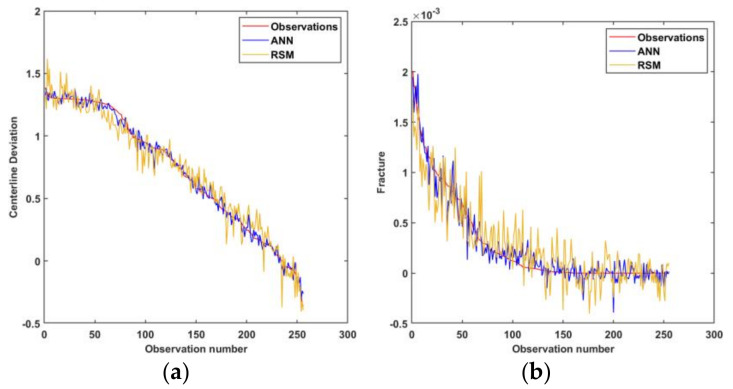
Model values for observations: (**a**) centerline deviation, (**b**) fracture objectives.

**Figure 16:**
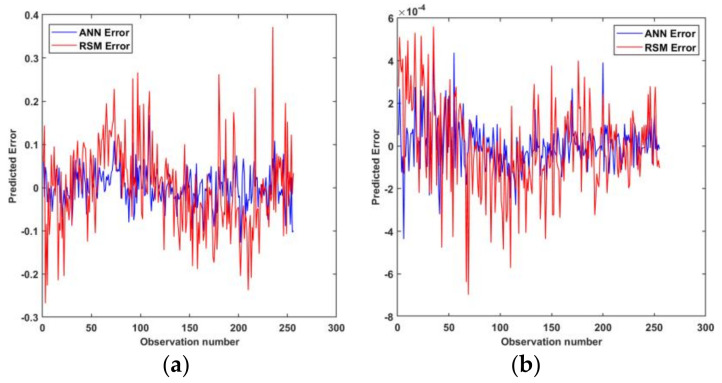
Prediction error for observations: (**a**) centerline deviation, (**b**) fracture objectives.

Statistical criteria were used to evaluate and compare the prediction capabilities of the obtained models. Figure 14 shows the predicted vs. observed diagrams for both models. Figure 15 shows the objective function values of the observations. Figure 16 shows a comparison of the prediction errors for both models. Table 4 lists the statistical features of the surrogate models. As can be seen from Figure 14, Figure 15 and Figure 16 and Table 4, the predictions of ANN are more accurate than those of RSM.

**Figure 17:**
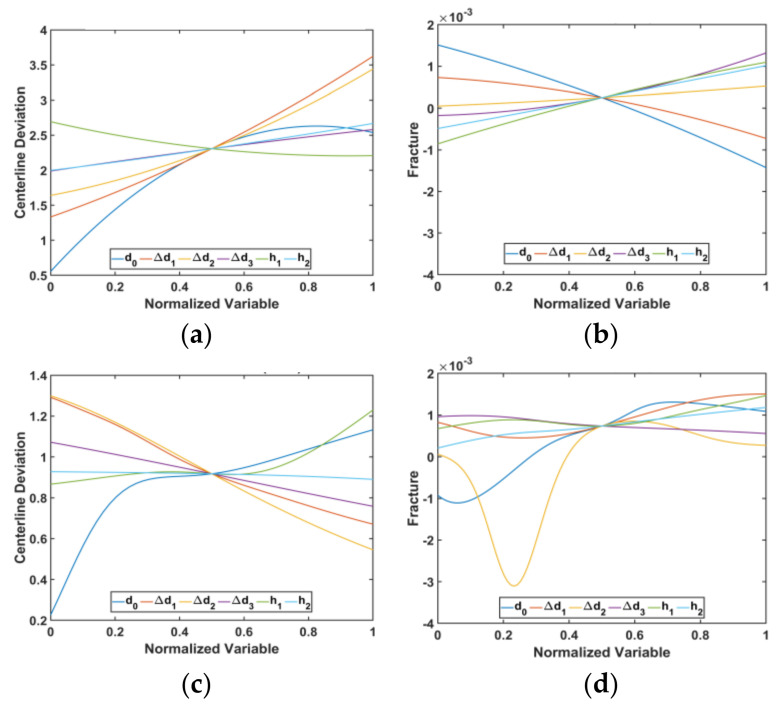
Variation of objective functions in each variable when other variables are fixed. RSM: (**a**) centerline deviation, (**b**) fracture; ANN: (**c**) centerline deviation, (**d**) fracture.

Figure 17 shows the variation of the objective functions for each variable when the other variables were fixed. As seen in Figure 17, the parameter $d_{0}$ has the most influence and directly affects the centerline deviation in both models; the centerline deviation increased with an increase in $d_{0}$. The gradient of change in the centerline deviation decreased at the end of the $d_{0}$ interval. In terms of fracture objectives, $d_{0}$ has the most influence on RSM, whereas $\Delta d_{1}$ has a higher effect than other variables in the ANN.

**Figure 18:**
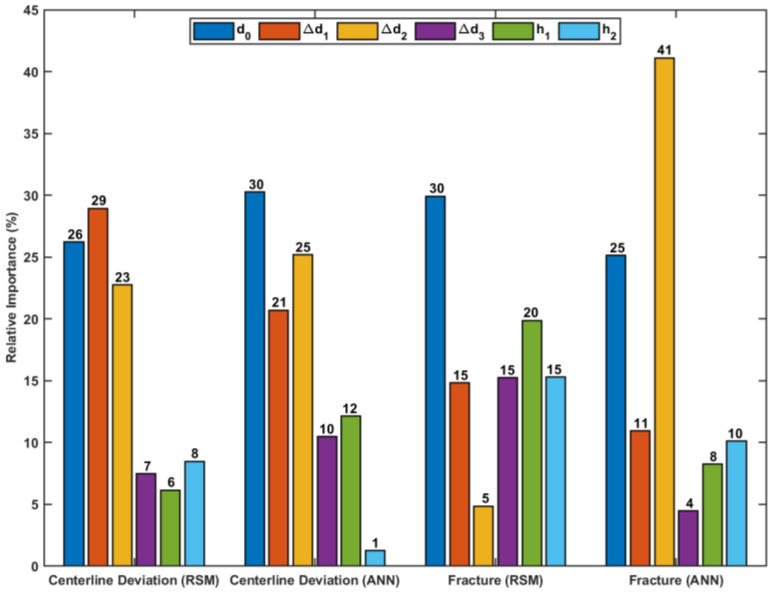
The relative importance of variables on objective functions.

Figure 18 shows the relative importance of variables on the objective functions. The relative importance was calculated using Equation (12).

(12) $$RI_{var}\left(\%\right)=\frac{Obj_{max}^{Var}-Obj_{min}^{Var}}{Obj_{max}^{overall}-Obj_{min}^{overall}}\times 100$$

The parameters $d_{0}$, $\Delta d_{1}$, and $\Delta d_{2}$ had more influence than that of other parameters, whereas the parameters $h_{2}$ and $\Delta d_{3}$ had the least effect. However, the parameter effects were different for the models and objectives. For example, $\Delta d_{2}$ had the most influence on fracture in the ANN.

The RSM used a quadratic polynomial with 28 coefficients, which were determined using Equation (7) in a single step. On the other hand, the ANN is a network with nine and eight neurons in the hidden layers for the fracture and centerline deviation objectives, respectively, trained with a gradient-based minimization algorithm. The ANN consisted of 73 and 65 coefficients (weights and biases) for the fracture and centerline deviation objectives, respectively, which showed higher nonlinearity than the RSM. Therefore, the effect of the parameters was more accurately modeled in the ANN than the RSM, as the statistical comparison of the two models confirmed the higher prediction accuracy of the ANN model.

### 4.2. Optimization Results

**Figure 19:**
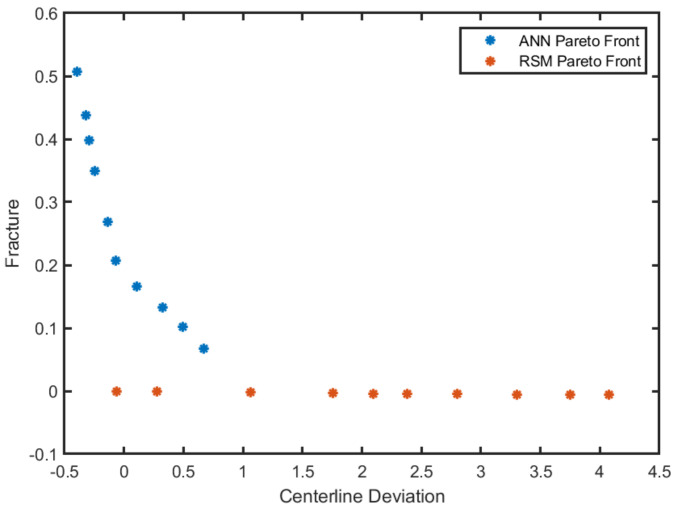
Optimized Pareto front: RSM, ANN.

By employing NSGA-II, a Pareto front was obtained for each model; Figure 19 shows the Pareto front achieved. Considering the design priorities, every point can be selected as an optimum in each front; the ANN shows better accuracy than RSM.

**Figure 20:**
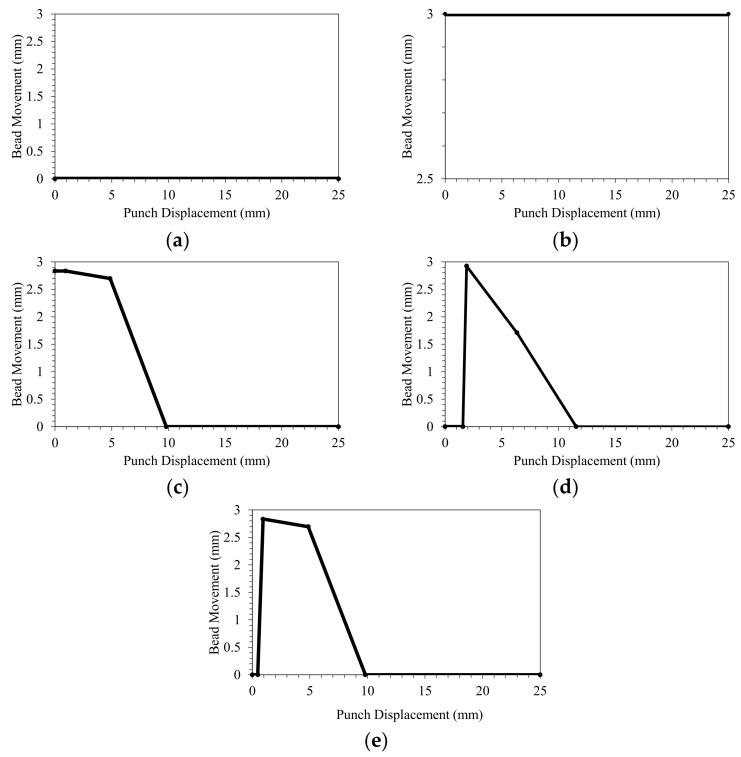
Drawbead trajectories of five cases: (**a**) no drawbead, (**b**) fixed drawbead, (**c**) adjustable drawbead (optimized with ANN, with initially raised bead), (**d**) adjustable drawbead (optimized with RSM), and (**e**) adjustable drawbead (optimized with ANN).

Figure 20 compares the drawbead trajectories of five cases: no drawbead, fixed drawbead, the adjustable drawbead (optimized with ANN, with initially raised bead), adjustable drawbead (optimized with RSM), and adjustable drawbead (optimized with ANN). 

**Figure 21:**
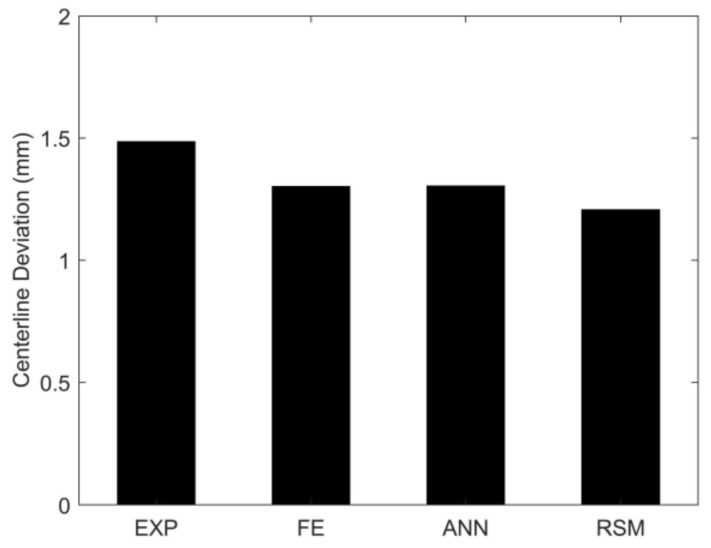
Comparison of the centerline deviation for the no drawbead case.

To compare the prediction capabilities of the FE model, the RSM, and the ANN, the centerline deviation was compared for the no drawbead case with the experiment [34]. As shown in Figure 21, the centerline deviations of the FE model, the RSM, and the ANN showed good agreements with the measurement.

**Figure 22:**
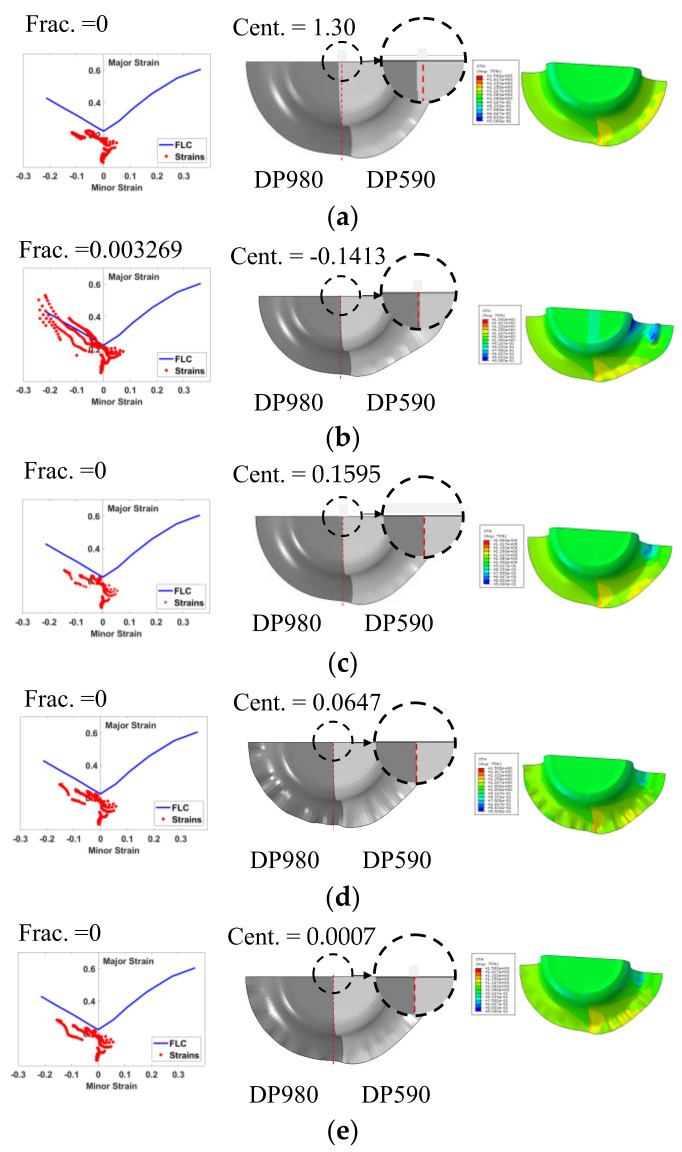
Forming limit diagram, deformed shape, and thickness distribution for five cases: (**a**) no drawbead, (**b**) fixed drawbead, (**c**) adjustable drawbead (optimized with ANN, with initially raised bead), (**d**) adjustable drawbead (optimized with RSM), and (**e**) adjustable drawbead (optimized with ANN).

Figure 22 shows the forming limit diagram of the five cases, deformed shape, and thickness distribution.

The results show that the proposed approach using ANN yielded the best results for both objectives; the ANN results were much more effective and accurate than the RSM. The fracture objective value is almost zero in both models, but the centerline deviation of the ANN (0.00076) is much smaller than the RSM optimum (0.064). As seen in Figure 21, the adjustable drawbead controlled the sheet flow, and consequently, centerline deviation was minimized compared to the no bead and the fully fixed bead cases.

The overall optimization results are summarized in Table 5, including the predicted objectives, FE calculated objectives, and prediction accuracy for the five cases. As discussed before, the delayed initiation of the adjustable drawbead ($d_{0}$) appears to be very effective, especially with respect to the centerline deviation. To investigate the effect of this parameter, the best optimum result (ANN optimum) was analyzed with the initially raised drawbead: the bead was positioned at a specific height before the start of deep drawing. This condition increased the centerline deviation dramatically from 0.00076 to 0.159516. It can be concluded that the increase in the bead height during the drawing process (not before the drawing) reduces the centerline deviation effectively.

## 5. Conclusions

In this study, an adjustable drawbead was introduced to improve the formability of the deep drawing of TWBs. The optimum bead movement was obtained using the multi-objective optimization algorithm (NSGA-II) together with the RSM and ANN to minimize the forming defects in the deep drawing of TWBs. The following conclusions were drawn:

1. The proposed procedure successfully determined the optimum movement of the adjustable drawbead to avoid fracture while minimizing the movement of the weld line.
2. The ANN was more accurate than the RSM in modeling a highly non-linear fracture objective function.
3. Among the six design variables of the drawbead movement, the displacement to the initiation of the bead ($d_{0}$) had the most influence on the fracture and centerline objective functions for both RSM and ANN models.
4. The design variables of the displacement to the initiation of the bead ($d_{0}$), the displacement to the first peak ($\Delta d_{1}$), and the displacement to the second peak ($\Delta d_{2}$) had a higher effect on centerline deviation than other parameters.
5. The displacement to the second peak ($\Delta d_{2}$) had the most relative importance in the fracture objective of the ANN, but it had the least importance in the RSM. This difference demonstrates the necessity of using an ANN with high nonlinearity.
6. A comparison of the optimum case (ANN) and the case with the initially raised bead showed that the delayed initiation of the drawbead is effective in reducing the movement of the weld centerline of the tailor-welded blank.

## Figures and Tables

**Table 1:** Chemical composition of the base sheets (wt %).

Material	C	Mn	Si	P	S
DP590	0.1	2	0.2	0.03	0.003
DP980	0.1	2.6	0.3	0.03	0.003

**Table 2:** Mechanical properties of the sheet blanks.

Material	DP590	DP980
Thickness, mm	1.0	1.0
Young’s modulus, GPa	210	210
Poisson’s ratio	0.3	0.3
Yield strength, MPa	382	849
Tensile strength, MPa	643	1058

**Table 3:** Parameters of trained ANNs.

Objective	Fracture	Centerline Deviation
Neurons in the input layer	3	3
Number of hidden layers	1	1
Neurons in the hidden layer	9	8
Neurons in the output layer	1	1
Training algorithm	Levenberg–Marquardt Back-Propagation	Levenberg–Marquardt Back-Propagation
Activation function (Hidden Layer)	Tansig	Tansig
Activation function (Output Layer)	Purelin	Purelin
Validation data fraction (%)	15	15
Test data fraction (%)	15	15

**Table 4:** Statistical features of surrogate models.

Objective	Model	MSE	RMSE	R
Fracture	RSM	4.3437 × 10^−8^	2.08414 × 10^−4^	0.883
Fracture	ANN	1.30063 × 10^−8^	1.14045 × 10^−4^	0.967
Centerline	RSM	9.86692 × 10^−3^	4.32202 × 10^−2^	0.978
Centerline	ANN	4.32203 × 10^−2^	9.93323 × 10^−2^	0.996

**Table 5:** Overall optimization summary.

Variable/Objective	No Bead	Fixed Bead	ANN Optimum (Initially Raised Bead)	RSM Optimum	ANN Optimum
*d*_0_ (mm)	0	0	0	1.566289	0.470124
Δ*d*_1_ (mm)	0	0	0	0.538199	0.466919
Δ*d*_2_ (mm)	0	25	3.921509	4.233494	3.921509
Δ*d*_3_ (mm)	0	0	4.959106	5.225806	4.959106
*h*_1_ (mm)	0	3	2.833374	2.923667	2.833374
*h*_2_ (mm)	0	3	2.695679	1.711225	2.695679
Fracture (predicted)	−0.060388	19.77237	0.2432341	−0.00015	0.20930
Fracture (FE)	0	0.003269	0	0	0
Centerline (predicted)	1.3041367	−0.256515	−0.043089	−0.06106	−0.06766
Centerline (FE)	1.3015800	−0.141269	0.159516	0.064793	0.00076

## Data Availability

The data presented in this study are available upon request from the corresponding author.
